# DA-Raf-Mediated Suppression of the Ras—ERK Pathway Is Essential for TGF-β1-Induced Epithelial—Mesenchymal Transition in Alveolar Epithelial Type 2 Cells

**DOI:** 10.1371/journal.pone.0127888

**Published:** 2015-05-21

**Authors:** Haruko Watanabe-Takano, Kazunori Takano, Masahiko Hatano, Takeshi Tokuhisa, Takeshi Endo

**Affiliations:** 1 Department of Biology, Graduate School of Science, Chiba University, Inage-ku, Chiba, Japan; 2 Department of Nanobiology, Graduate School of Advanced Integral Science, Chiba University, Inage-ku, Chiba, Japan; 3 Biomedical Research Center, Chiba University, Chuo-ku, Chiba, Japan; 4 Department of Developmental Genetics, Graduate School of Medicine, Chiba University, Chuo-ku, Chiba, Japan; 5 Japan Society for the Promotion of Science (JSPS), Chiyoda-ku, Tokyo, Japan; Helmholtz Zentrum München, GERMANY

## Abstract

Myofibroblasts play critical roles in the development of idiopathic pulmonary fibrosis by depositing components of extracellular matrix. One source of lung myofibroblasts is thought to be alveolar epithelial type 2 cells that undergo epithelial–mesenchymal transition (EMT). Rat RLE-6TN alveolar epithelial type 2 cells treated with transforming growth factor-β1 (TGF-β1) are converted into myofibroblasts through EMT. TGF-β induces both canonical Smad signaling and non-canonical signaling, including the Ras-induced ERK pathway (Raf–MEK–ERK). However, the signaling mechanisms regulating TGF-β1-induced EMT are not fully understood. Here, we show that the Ras–ERK pathway negatively regulates TGF-β1-induced EMT in RLE-6TN cells and that DA-Raf1 (DA-Raf), a splicing isoform of A-Raf and a dominant-negative antagonist of the Ras–ERK pathway, plays an essential role in EMT. Stimulation of the cells with fibroblast growth factor 2 (FGF2), which activated the ERK pathway, prominently suppressed TGF-β1-induced EMT. An inhibitor of MEK, but not an inhibitor of phosphatidylinositol 3-kinase, rescued the TGF-β1-treated cells from the suppression of EMT by FGF2. Overexpression of a constitutively active mutant of a component of the Ras–ERK pathway, i.e., H-Ras, B-Raf, or MEK1, interfered with EMT. Knockdown of DA-Raf expression with siRNAs facilitated the activity of MEK and ERK, which were only weakly and transiently activated by TGF-β1. Although DA-Raf knockdown abrogated TGF-β1-induced EMT, the abrogation of EMT was reversed by the addition of the MEK inhibitor. Furthermore, DA-Raf knockdown impaired the TGF-β1-induced nuclear translocation of Smad2, which mediates the transcription required for EMT. These results imply that intrinsic DA-Raf exerts essential functions for EMT by antagonizing the TGF-β1-induced Ras–ERK pathway in RLE-6TN cells.

## Introduction

Idiopathic pulmonary fibrosis (IPF) is a chronic, progressive, irreversible, and usually lethal lung disease characterized by interstitial fibrosis of unknown pathogenesis [1–3]. A growing body of evidence indicates that the disease is the result of a fibrotic response driven by abnormally activated alveolar epithelial cells (AECs). These cells produce mediators that induce the formation of myofibroblast foci through the proliferation and activation of resident fibroblasts, attraction of circulating fibrocytes, and stimulation of epithelial—mesenchymal transition (EMT). The myofibroblast foci deposit excessive amounts of extracellular matrix (ECM) components such as collagen and fibronectin, resulting in scarring and destruction of the lung architecture, leading to IPF. Several recent studies have supported the notion that myofibroblasts or fibroblasts generated by EMT of type 2 AECs (AEC2s) are, at least in part, responsible for pulmonary fibrosis [4–8]. These studies use human cells and tissues from IPF patient lungs; rodent lung models treated with bleomycin or transforming growth factor-β1 (TGF-β1); and rodent primary cultured AEC2s or rat AEC2 cell line RLE-6TN (RLE) cells treated with TGF-β1, endothelin-1 (ET-1), or cultured on fibronectin. EMT by the ET-1 treatment or culture on fibronectin is mediated by the induction of TGF- β 1 signaling.

TGF-βis generally recognized as a central mediator of the fibrotic response in physiological tissue repair and in many fibrotic diseases by inducing EMT, activating fibroblasts, and promoting synthesis of the ECM components [9–13]. TGF-β signaling is induced through type I and type II protein Ser/Thr kinase receptors (TβRI and TβRII) [14]. TGF-β binding induces activating phosphorylation of dimeric TβRIs by dimeric TβRIIs. Subsequently, the activated TβRIs recruit and phosphorylate receptor-regulated Smad (R-Smad), Smad2/3. Phosphorylated Smad2/3 dissociates from the receptors and binds to co-Smad, Smad4. The activated Smad2/3–Smad4 heterotrimeric complex translocates into the nucleus and regulates the transcription of specific target genes together with transcriptional coactivators or corepressors.

Besides canonical Smad signaling, TGF-β induces non-canonical, non-Smad signaling including the Ras—ERK pathway, TRAF6—TAK1—JNK/p38 MAPK, RhoA/Cdc42, and PI3K—Akt signaling [15–17]. Combination or crosstalk of these non-Smad signaling pathways or between Smad and non-Smad signaling produces diverse biological responses of TGF-β, such as cell proliferation, differentiation, growth arrest, apoptosis, and EMT. The Ras-induced ERK pathway (Raf—MEK—ERK) is typically activated by growth factors through their receptor tyrosine kinases (RTKs), which mobilize adaptor proteins, such as Shc and Grb2, and guanine nucleotide exchange factors (GEFs) like Sos. The RTK-mediated activation of a specific GEF activates the small GTPase Ras (H-, K-, and N-Ras) via GTP loading. Association of the activated Ras with Raf family proteins (B- and C-Raf) leads to MEK and ERK activation through sequential phosphorylation [18–20]. Similarly to RTKs, activated TβRIs recruit and directly phosphorylate ShcA on Tyr as well as Ser [21]. Although the Tyr kinase activity of TβRIs is much lower than that of RTKs, this phosphorylation of ShcA triggers association with Grb2 and Sos, thereby activating the Ras—ERK pathway.

The effects of the Ras—ERK pathway on TGF-β1-induced EMT are distinct among different cell types. Active Ras and the ERK pathway are required for TGF-β1-induced EMT in human keratinocytes [22, 23]. In addition, active Ras or Raf induces EMT cooperatively with TGF-β1 in canine kidney epithelial cells and mouse mammary epithelial cells [24, 25]. In contrast, the Ras—ERK pathway interferes with TGF-β1-induced, Smad signaling-mediated EMT in primary cultured AECs and AEC cell lines [26, 27]. The growth factor-induced Ras—ERK pathway impedes EMT in AECs through the expression of the Smad signaling inhibitor Smad7, the nuclear export of Smad7 and the E3 ubiquitin ligase Smurf1, or the dephosphorylation of Smad2. However, it remains to be clarified whether the TGF-β1-induced Ras—ERK pathway is required or needs to be silenced for EMT induction in AECs.

We have found DA-Raf1 (DA-Raf), which intrinsically antagonizes the Ras—ERK pathway [28, 29]. DA-Raf is generated by alternative splicing from the *Araf* gene and contains the Ras-binding domain but lacks the kinase domain responsible for activation of the ERK pathway. On the basis of this structure, DA-Raf binds to active Ras and M-Ras and interferes with the Ras—ERK pathway. Consequently, DA-Raf serves as a positive regulator of myogenic differentiation and lung alveolarization, both of which are prevented by the Ras—ERK pathway. Furthermore, exogenously expressed DA-Raf suppresses oncogenic Ras-induced cellular transformed phenotypes and tumorigenicity in cell-transplanted mice.

To clarify the signaling mechanisms of TGF-β1-induced EMT in RLE cells, we have addressed the question of whether DA-Raf participates in the induction of EMT by regulating the Ras—ERK pathway. Here, we show that the Ras—ERK pathway negatively regulates the TGF-β1-induced EMT in RLE cells and that endogenous DA-Raf plays essential roles in EMT by suppressing the TGF-β1-induced Ras—ERK pathway.

## Results

### Sustained activation of the Ras—ERK pathway by FGF2 inhibits TGF-β1-induced EMT in RLE cells

**Fig 1:**
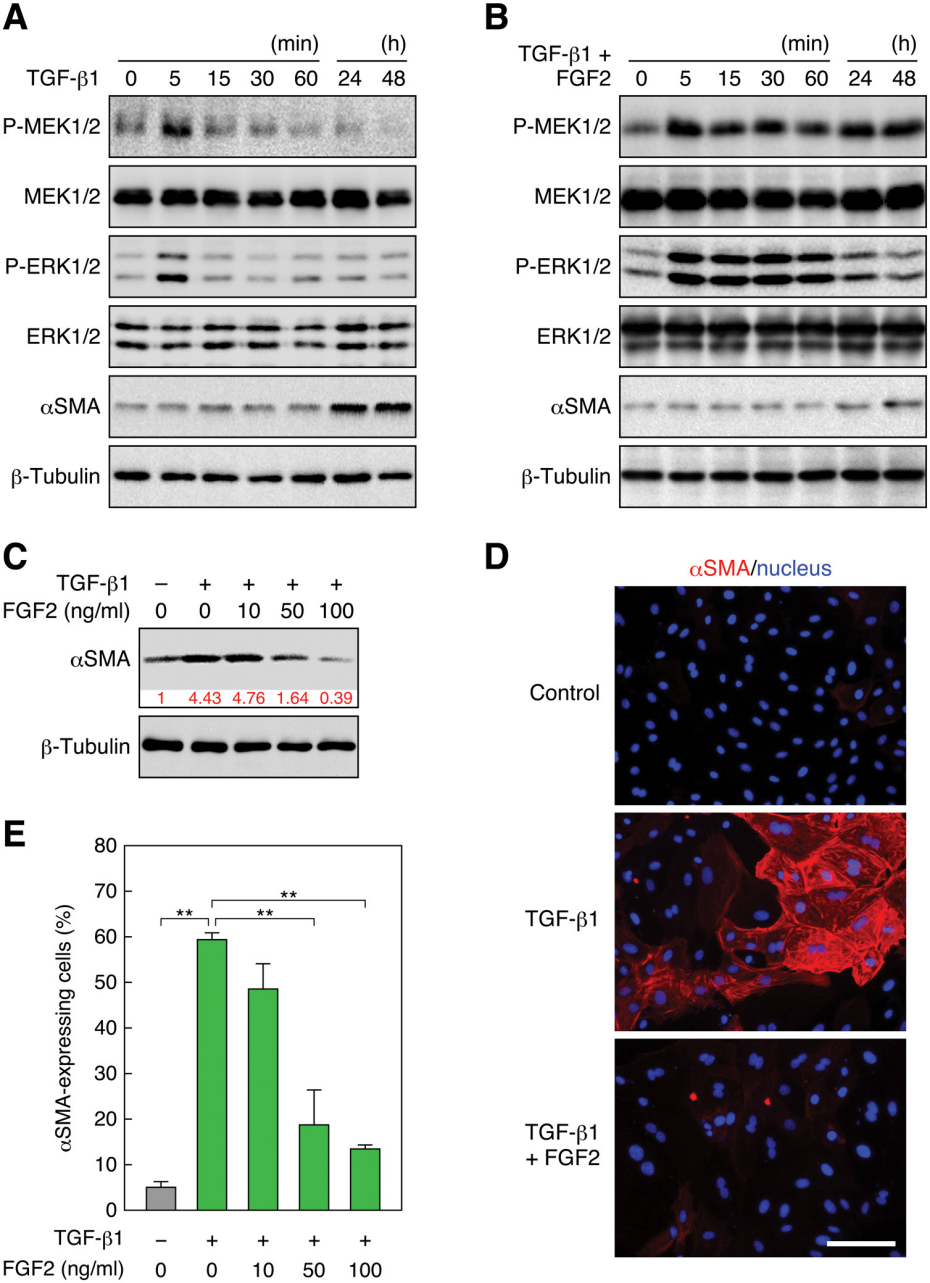
FGF2 induces sustained activation of the Ras—ERK pathway and inhibits TGF-β1-induced EMT. (A) Transient phosphorylation of MEK and ERK and induction of αSMA expression by TGF-β1 stimulation. RLE cells were stimulated with 0.5 ng/ml TGF-β1. The levels of MEK, phospho (P)-MEK, ERK, P-ERK, and αSMA, as well as β -tubulin as a standard, were analyzed by immunoblotting. (B) Sustained phosphorylation of MEK and ERK and inhibition of αSMA expression by FGF2 stimulation. RLE cells were stimulated with 100 ng/ml FGF2 in combination with 0.5 ng/ml TGF-β1. (C) A dose-dependent reduction of the TGF- β 1-induced αSMA protein level by FGF2 stimulation. RLE cells were stimulated with the indicated concentrations of FGF2 together with 0.5 ng/ml TGF- β 1 for 48 h. The relative intensity of αSMA band is indicated under the blot. (D) Induction of αSMA expression by TGF- β 1 and suppression of the expression by FGF2. RLE cells were stimulated with 0.5 ng/ml TGF- β 1 or with 100 ng/ml FGF2 along with TGF- β 1 for 48 h. αSMA expression and localization was detected by immunofluorescent staining with the Cy3—anti-αSMA mAb (red) as well as nuclear staining with Hoechst 33258 (blue). Scale bar, 50 μm. (E) A dose-dependent reduction of the ratio of TGF- β 1-induced αSMA-expressing cells by FGF2 stimulation. αSMA-expressing cells were detected as in (D). The values are means ± SD of 3 independent experiments. **, *P* < 0.01 by *t* test.

TGF-β activates both Smad signaling and the Ras—ERK pathway. Although Smad signaling is indispensable for TGF-β-induced EMT, it has remained controversial whether the Ras—ERK pathway is required for or interferes with EMT. To determine the role of the Ras—ERK pathway in EMT in RLE cells, first we analyzed the effects of fibroblast growth factor 2 (FGF2) on TGF-β1-induced EMT. When RLE cells were treated with TGF-β1, the phosphorylation levels of both MEK1/2 and ERK1/2 were elevated within 5 min (Fig 1A). Both the phosphorylation levels declined to basal levels by 15 min, however, and the reduced levels were retained for at least 48 h. In contrast, when the cells were stimulated with 100 ng/ml FGF2 together with TGF- β 1, the phosphorylation levels of both MEK1/2 and ERK1/2 increased within 5 min, and these levels were sustained for more than 24 h (Fig 1B).

Next, to estimate the transitional state from AEC2s to myofibroblasts in RLE cells, we analyzed the expression of α-smooth muscle actin (αSMA), a marker protein of myofibroblasts [30]. Treatment of the cells with TGF-β1 did not affect the amount of αSMA for at least 60 min but highly induced αSMA during 24–48 h after the treatment (Fig 1A). On the other hand, stimulation of the cells with FGF2 along with TGF- β 1 suppressed the induction of αSMA even during the 24–48 h period (Fig 1B). FGF2 inhibited the TGF- β 1-induced αSMA expression in a dose-dependent manner, i.e., 10 ng/ml FGF2 did not suppress the αSMA expression, but 50 ng/ml FGF2 reduced the expression to the basal level, and 100 ng/ml FGF2 further reduced the expression (Fig 1C). Immunofluorescence microscopy located the TGF- β 1-induced αSMA to stress fibers in RLE cells (Fig 1D), indicating that the expressed αSMA carries out the contractile function of myofibroblasts [30]. Addition of FGF2 dose-dependently decreased the ratio of αSMA-expressing cells (Fig 1E), and 100 ng/ml FGF2 prominently reduced the ratio of αSMA-expressing cells (Fig 1D and 1E). These results imply that the low levels of transient activation of MEK and ERK, which are brought about by TGF- β 1 treatment, do not inhibit EMT, but that the FGF2-induced high levels of sustained activation of MEK and ERK suppress TGF- β 1-induced EMT in RLE cells.

### Activation of the Ras—ERK pathway but not PI3K—Akt signaling inhibits TGF-β1-induced EMT in RLE cells

**Fig 2:**
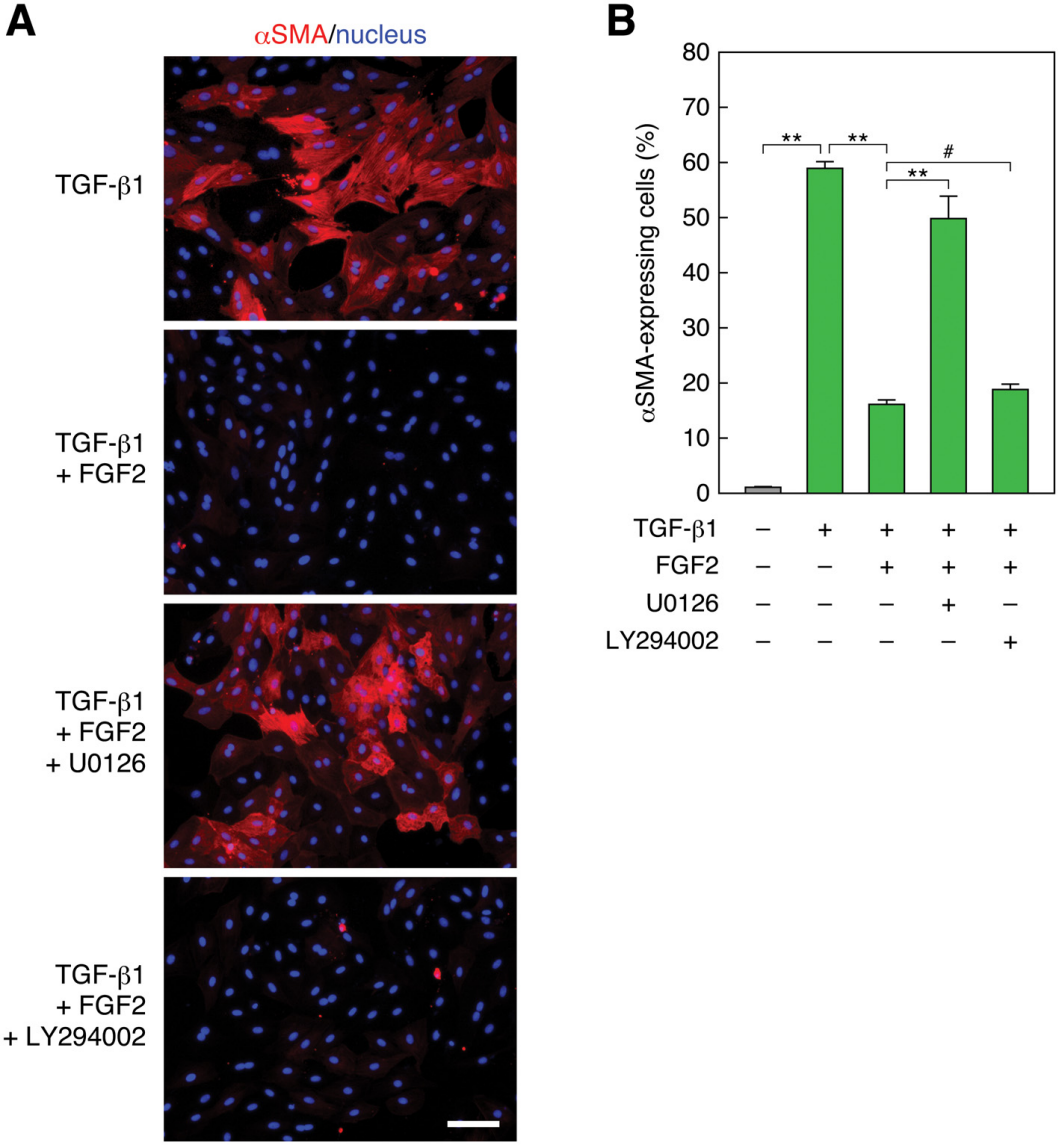
Inhibition of MEK but not PI3K recovers TGF-β1-induced and FGF2-suppressed EMT. (A) Recovery of FGF2-suppressed αSMA expression by MEK inhibition but not by PI3K inhibition. RLE cells were pretreated with 10 μM of the MEK inhibitor U0126 or the PI3K inhibitor LY294002 for 30 min. Then they were stimulated with 0.5 ng/ml TGF-β1 along with 100 ng/ml FGF2 for 48 h. αSMA expression (red) and nuclei (blue) were detected as described in Fig 1 legend. Scale bar, 50 μm. (B) The ratio of αSMA-expressing cells in the analysis of (A). The values are means ± SD of 3 independent experiments. **, *P* < 0.01; #, *P* > 0.05 (not significant) by *t* test.

Growth factors activate not only the Ras—ERK pathway but also PI3K—Akt signaling [31]. TGF- β also induces both the Ras—ERK pathway and PI3K—Akt signaling as non-Smad signaling [16, 17]. To determine whether only the Ras—ERK pathway or whether both the Ras—ERK pathway and PI3K—Akt signaling are responsible for the FGF2-caused suppression of EMT, we analyzed the effects of inhibitors of these signaling pathways in RLE cells. Treatment of the cells with the MEK inhibitor U0126 recovered the stress fiber-associated αSMA expression that was suppressed by FGF2 stimulation (Fig 2A). U0126 treatment also restored the ratio of αSMA-expressing cells nearly to the ratio induced by TGF- β 1 (Fig 2B). In contrast, treatment with the PI3K inhibitor LY294002 had no detectable effect on FGF2-caused suppression of αSMA expression (Fig 2A and 2B). Consequently, FGF2-induced strong and sustained activation of the Ras—ERK pathway (see Fig 1B) but not PI3K—Akt signaling hinders EMT. We also tried to evaluate the effects of TGF- β 1-induced weak and transient activation of the Ras—ERK pathway and PI3K—Akt signaling on EMT by adding U0126 and LY294002, respectively. However, addition of either inhibitor together with TGF- β 1 treatment resulted in extensive apoptosis. Thus, we could not estimate the effects on these signaling pathways (data not shown, see discussion).

**Fig 3:**
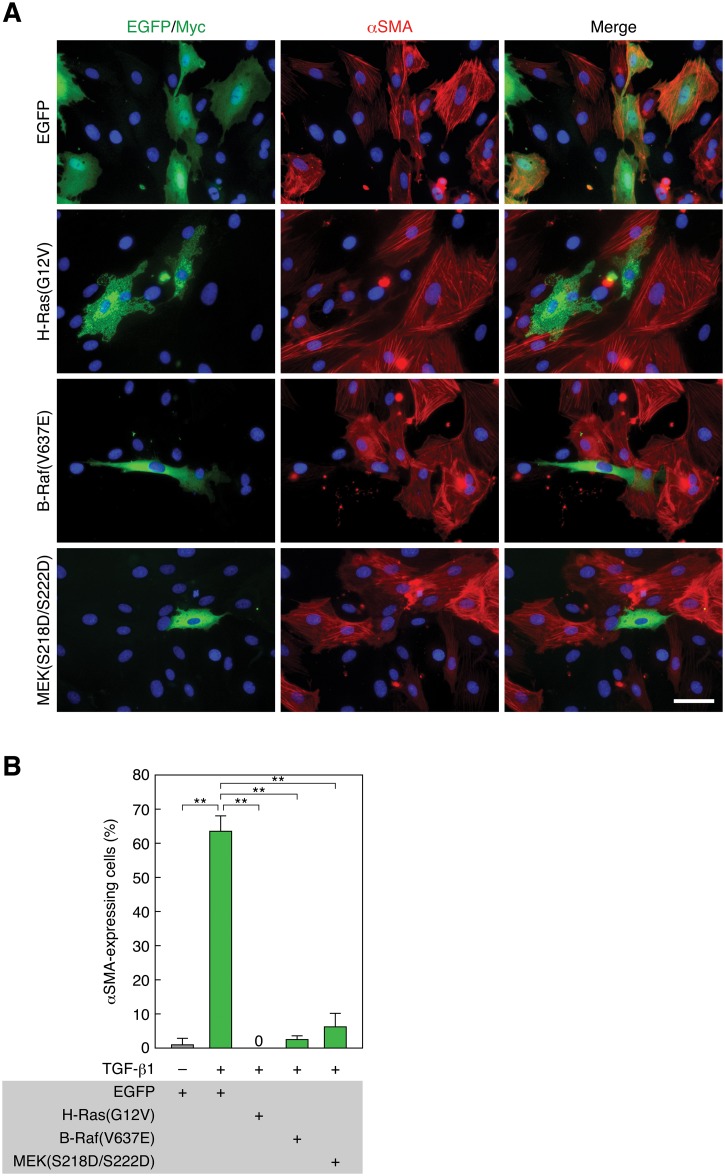
Constitutively active H-Ras, B-Raf, or MEK1 suppresses TGF-β1-induced EMT. (A) Suppression of TGF-β1-induced EMT by constitutively active H-Ras, B-Raf, and MEK1. RLE cells were transfected with Myc-tagged H-Ras(G12V), EGFP-tagged B-Raf(V637E), EGFP—MEK1(S218D/S222D), or EGFP expression vector. Twenty-four hours after the transfection, they were treated with 0.5 ng/ml TGF-β1 for 48 h. αSMA expression (red) and nuclei (blue) were detected as described in Fig 1 legend. Myc- and EGFP-tagged proteins were detected by anti-Myc pAb and anti-GFP pAb staining, respectively (green). Scale bar, 20 μm. (B) The ratio of αSMA-expressing cells in the analysis of (A). The values are means ± SD of 3 independent experiments. **, *P* < 0.01 by *t* test.

Next, we analyzed the effects of constitutively active mutants of Ras—ERK pathway components on EMT. When we transfected RLE cells with a constitutively active oncogenic mutant of H-Ras(G12V) (Gly12 is converted to Val) and treated with TGF-β1 for 48 h, cells expressing H-Ras(G12V) never expressed αSMA, whereas cells lacking H-Ras(G12V) expression showed αSMA expression and its localization to stress fibers (Fig 3A and 3B). Transfection of B-Raf(V637E), a murine constitutively active oncogenic mutant corresponding to human B-Raf(V600E) [32], also strikingly suppressed αSMA expression (Fig 3A and 3B). Similarly, transfection of MEK1(S218D/S222D), a constitutively active pseudophosphorylation mutant [33], noticeably suppressed αSMA expression (Fig 3A and 3B). These results corroborate the above notion that strong and sustained activation of the Ras—ERK pathway blocks EMT in RLE cells.

### Expression of DA-Raf is required for TGF-β1-induced EMT in RLE cells

**Fig 4:**
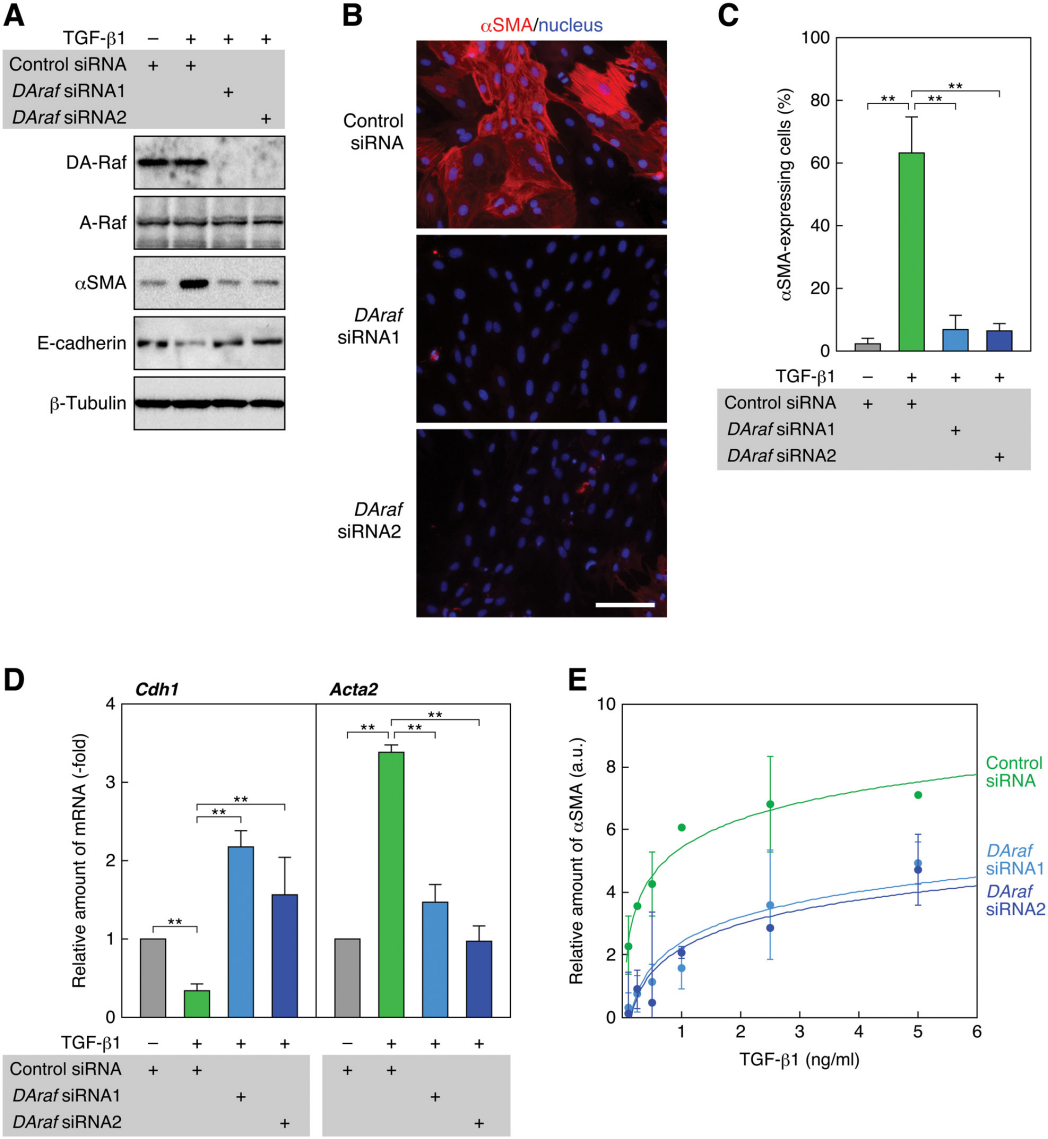
Knockdown of DA-Raf abrogates TGF-β1-induced EMT. (A) Suppression of TGF-β1-induced αSMA expression by knockdown of DA-Raf with *DAraf* siRNAs. RLE cells were transfected with *DAraf* siRNAs as well as a control siRNA. Twenty-four hours after the transfection, they were treated with 0.5 ng/ml TGF-β1 for 48 h. The levels of DA-Raf, A-Raf, αSMA, and E-cadherin, as well as β-tubulin as a standard, were analyzed by immunoblotting. (B) Suppression of TGF-β1-induced αSMA expression with *DAraf* siRNAs. RLE cells were transfected with *DAraf* siRNAs and treated with 0.5 ng/ml TGF-β1. αSMA expression (red) and nuclei (blue) were detected by fluorescence microscopy. Scale bar, 50 μm. (C) The ratio of αSMA-expressing cells in the analysis of (B). The values are means ± SD of 3 independent experiments. **, *P* < 0.01 by *t* test. (D) Elevation of the TGF-β1-suppressed *Cdh1* (E-cadherin) mRNA level and suppression of the TGF-β1-induced *Acta2* (αSMA) mRNA level with *DAraf* siRNAs. RLE cells were transfected with *DAraf* siRNAs and treated with TGF-β1 for 48 h. Relative levels of *Cdh1* and *Acta2* mRNAs normalized to the *Actb* (β-actin) mRNA level were determined by real-time PCR. The values are means ± SD of 3 independent experiments. **, *P* < 0.01 by *t* test. (E) Dose-dependent induction of αSMA expression by TGF-β1 and its suppression by *DAraf* siRNAs. RLE cells were transfected with *DAraf* siRNAs and treated with 0.1–5 ng/ml TGF-β1 for 48 h. The intensities of αSMA and β-tubulin bands on immunoblots were analyzed by densitometry. The graph shows the ratio of αSMA to β-tubulin band intensity against TGF-β1 concentration. The values are means ± SD of 3 independent experiments. a.u., arbitrary units.

Although FGF2-induced strong and sustained activation of the Ras—ERK pathway hindered EMT, TGF-β1-induced weak and transient activation of the Ras—ERK pathway did not affect EMT (see Fig 1A–1E). Thus, regulatory mechanisms of the weak and transient activation of the Ras—ERK pathway induced by TGF-β1 need to be elucidated. Accordingly, we addressed the question of whether DA-Raf, an intrinsic antagonist of the Ras—ERK pathway [28, 29], negatively regulates the TGF-β1-induced Ras—ERK pathway to a level that does not suppress EMT. RLE cells highly expressed DA-Raf regardless of treatment with TGF-β1 (Fig 4A). To determine whether DA-Raf participates in EMT, we knocked down DA-Raf expression by RNA interference (RNAi) using two distinct small interfering RNAs (siRNAs) of *DAraf*. Both these siRNAs target the 3’ untranslated region of *DAraf* mRNA because this is the only region not included in *Araf* mRNA, which is generated by alternative splicing of common *Araf/DAraf* pre-mRNA [28]. Transfection of either of these two siRNAs in RLE cells strikingly reduced the level of endogenous DA-Raf protein, whereas neither affected the A-Raf protein level (Fig 4A). Thus, these siRNAs specifically knocked down DA-Raf expression without showing off-target effects on A-Raf expression. The knockdown of DA-Raf strongly suppressed TGF-β1-induced elevation of αSMA expression to a level comparable to that of the control cells without TGF-β1 treatment (Fig 4A). In addition, immunofluorescent staining of αSMA showed that the introduction of either of these two siRNAs strikingly reduced the number of αSMA-expressing cells induced by TGF-β1 treatment (Fig 4B and 4C). Since EMT in RLE cells is accompanied by a decrease in epithelial cell marker E-cadherin as well as an increase in myofibroblast marker αSMA [27], we also analyzed the effect of DA-Raf knockdown on E-cadherin expression. TGF-β1 treatment reduced the E-cadherin protein level, whereas the level was hardly affected in *DAraf* siRNA-introduced cells (Fig 4A).

Next, we analyzed the effect of DA-Raf knockdown during TGF-β1 treatment on the levels of *Cdh1* (E-cadherin) and *Acta2* (αSMA) mRNAs by quantitative real-time RCR. The *Cdh1* mRNA level was reduced by 48 h after TGF-β1 treatment (Fig 4D). In contrast, expression of either of the *DAraf* siRNAs interfered with the reduction of *Cdh1* mRNA and elevated the *Cdh1* mRNA level in comparison with that in control siRNA-expressing cells (Fig 4D). Thus, DA-Raf seems to antagonize epithelial cell properties in RLE cells. On the other hand, the *Acta2* mRNA level was increased by TGF-β1 treatment, and introduction of either of the *DAraf* siRNAs abrogated this increase (Fig 4D). This result is consistent with those of immunoblotting and immunofluorescence analyses (Fig 4A–4C). Taken together, these results indicate that DA-Raf is required for the induction of EMT by TGF-β1 in RLE cells.

Further, we evaluated the effect of DA-Raf on the αSMA expression induced by various concentrations of TGF-β1 by quantifying the αSMA level on immunoblots. TGF-β1 even at a low concentration (0.1 ng/ml) caused αSMA expression and dose-dependently facilitated αSMA expression at least to the concentration of 5 ng/ml (Fig 4E). Transfection of either of the *DAraf* siRNAs effectively reduced the αSMA level at any TGF-β1 concentration (Fig 4E). Accordingly, intrinsic DA-Raf is likely to maximally accelerate EMT induced by TGF-β1 at any concentrations examined.

### Suppression of the Ras—ERK pathway by DA-Raf is responsible for TGF-β1-induced EMT in RLE cells

**Fig 5:**
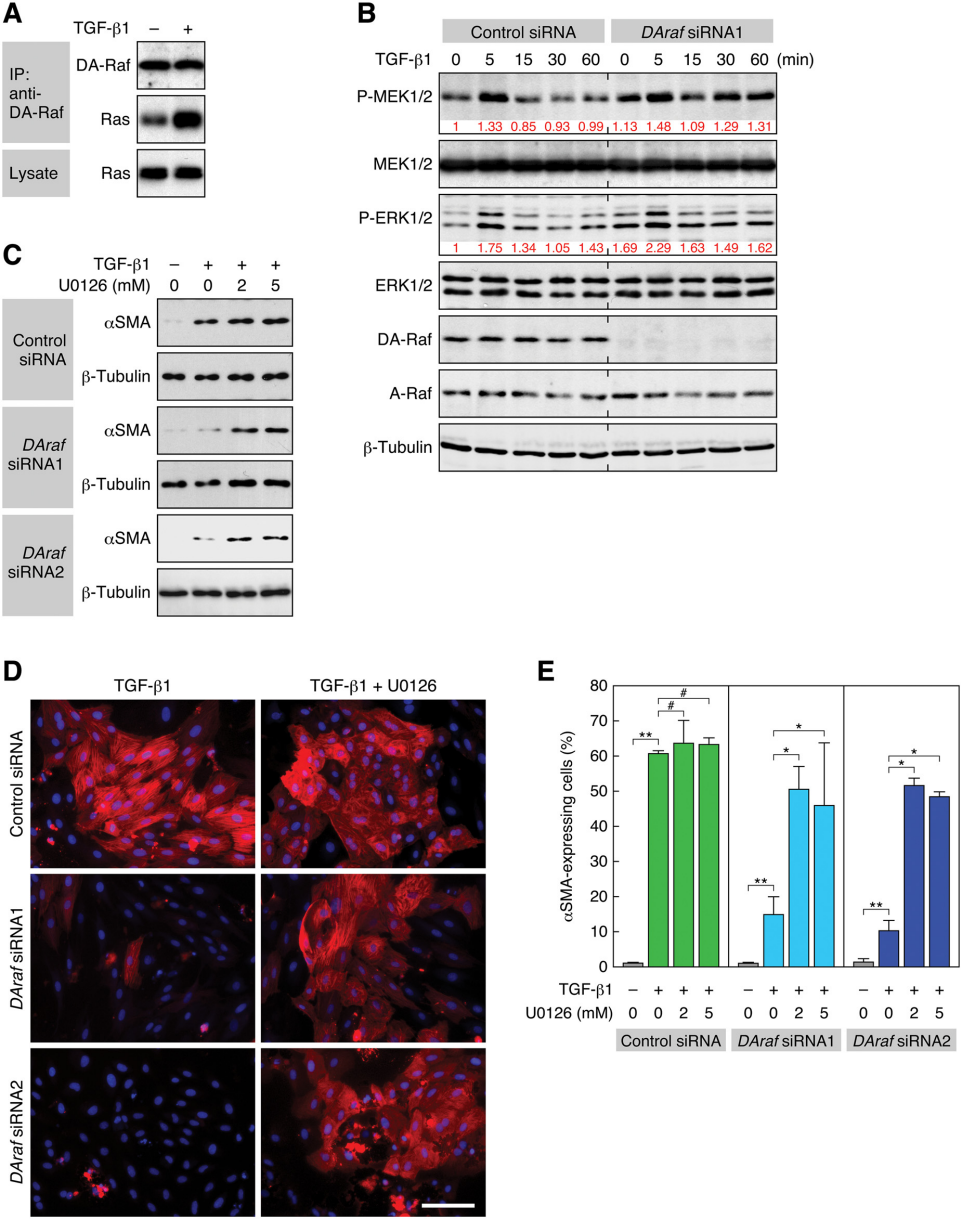
Suppression of the ERK pathway by DA-Raf is required for TGF-β1-induced EMT. (A) Induction of the binding of DA-Raf to Ras by TGF-β1 stimulation. The binding was analyzed by a coimmunoprecipitation assay. RLE cells were treated with 0.5 ng/ml TGF-β1 for 5 min. DA-Raf was immunoprecipitated with anti-DA-Raf pAb, and coprecipitated Ras was detected by immunoblotting with pan-Ras mAb. (B) Elevation of the phosphorylation levels of MEK and ERK by DA-Raf knockdown. RLE cells were transfected with *DAraf* siRNA1 as well as the control siRNA and treated with 0.5 ng/ml TGF-β1 for the indicated time. The levels of MEK, P-MEK, ERK, P-ERK, DA-Raf, A-Raf, and β-tubulin were analyzed by immunoblotting. The relative intensities of P-MEK1/2 and P-ERK1/2 bands are indicated under their blots. (C) Recovery of *DAraf* siRNA-blocked αSMA expression with U0126. RLE cells were transfected with *DAraf* siRNA1 or siRNA2 and then treated with 2 or 5 μM U0126 and 0.5 ng/ml TGF-β1 for 48 h. The level of αSMA, as well as β-tubulin as a standard, was analyzed by immunoblotting. (D) Recovery of *DAraf* siRNA-impaired αSMA expressing cells with U0126. RLE cells were transfected with *DAraf* siRNA1 or siRNA2 and then treated with 5 μM U0126 and 0.5 ng/ml TGF-β1 for 48 h. αSMA expression (red) and nuclei (blue) were detected. Scale bar, 50 μm. (E) The ratio of αSMA-expressing cells in the analysis of (D). The values are means ± SD of 3 independent experiments. *, *P* < 0.05; **, *P* < 0.01; #, *P* > 0.05 (not significant) by *t* test.

DA-Raf binds to active Ras and suppresses the ERK pathway. In consequence, intrinsic DA-Raf serves as a positive regulator of myogenic differentiation [28] and lung alveolarization [29], both of which are prevented by the Ras—ERK pathway. Accordingly, we examined whether DA-Raf is required for TGF-β1-induced EMT through suppressing the Ras—ERK pathway. A coimmunoprecipitation assay showed that stimulation of RLE cells with TGF-β1 induced the binding of DA-Raf to Ras (Fig 5A). Thus, we analyzed the phosphorylation status of MEK1/2 and ERK1/2 in the control and DA-Raf knockdown cells. Endogenous DA-Raf as well as A-Raf was expressed at a constant level for 60 min during TGF-β1 stimulation in the control cells (Fig 5B). In these cells, the phosphorylation levels of both MEK1/2 and ERK1/2 were transiently elevated at around 5 min after TGF-β1 stimulation and then declined to basal levels by 15 min. On the other hand, their phosphorylation levels were higher in DA-Raf knockdown cells than in the control cells at every time point for at least 60 min (Fig 5B). These results suggest that the binding of DA-Raf to Ras, which is activated by TGF-β1 stimulation, interferes with the activation of MEK and ERK in RLE cells.

Subsequently, we analyzed the effect of DA-Raf knockdown together with inhibition of the ERK pathway on TGF-β1-induced EMT. The expression level of αSMA in TGF-β1-stimulated RLE cells was hardly affected by treatment with U0126, which inhibits the ERK pathway (Fig 5C). On the other hand, although the level of αSMA in TGF-β1-stimulated cells was markedly reduced by the knockdown of DA-Raf with the *DAraf* siRNAs, treatment of the knockdown cells with U0126 recovered the αSMA level (Fig 5C). Immunofluorescent detection of αSMA-expressing cells also showed that treatment of the TGF-β1-stimulated cells with U0126 did not affect the level of αSMA expression or the ratio of αSMA-expressing cells (Fig 5D and 5E). On the other hand, knockdown of DA-Raf with either of the siRNAs notably reduced the level of αSMA expression and the ratio of αSMA-expressing cells induced by the TGF-β1 treatment (Fig 5D and 5E). However, treatment of the DA-Raf knockdown cells with U0126 compensated for this reduction (Fig 5D and 5E). Taken together, these results imply that intrinsic DA-Raf plays an essential role in TGF-β1-induced EMT in RLE cells by suppressing the Ras—ERK pathway.

**Fig 6:**
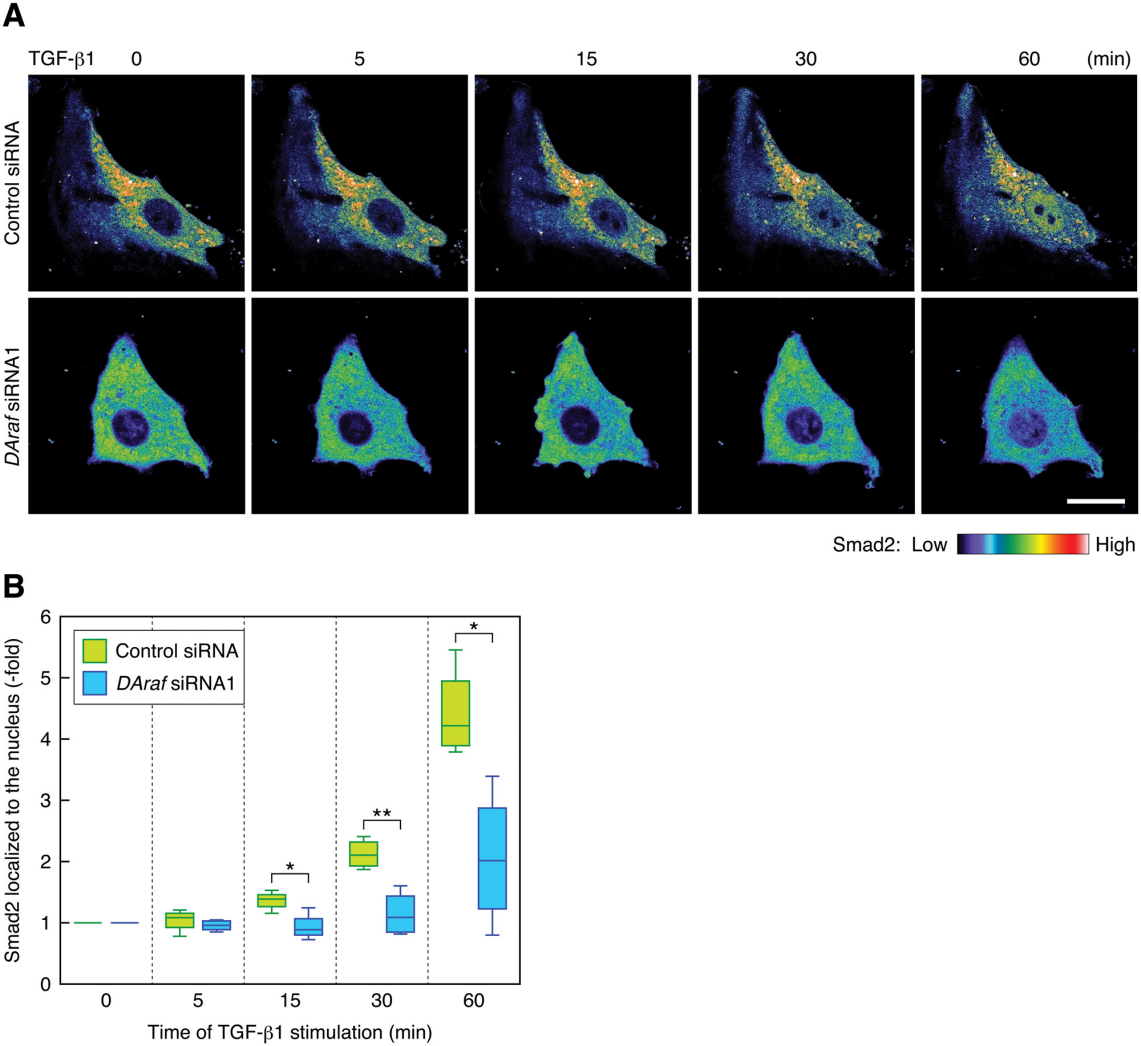
Knockdown of DA-Raf hinders the nuclear translocation of Smad2 induced by TGF-β1. (A) Live cell images of the localization of Smad2. mCherry—Smad2-expressing RLE cells transfected with the control siRNA or *DAraf* siRNA1 were stimulated with 0.5 ng/ml TGF-β1 for the indicated time. The color indicator shows fluorescence intensity of mCherry—Smad2. Scale bar, 20 μm. (B) The degree of mCherry—Smad2 localization in the nucleus in the analysis of (A). Smad2 localized to the nucleus was calculated from the nuclear/cytoplasmic ratio of mCherry—Smad2 intensity. The box plot represents the data of 4 independent live cell images. *, *P* < 0.03; **, *P* < 0.005 by *t* test.

Furthermore, to confirm the involvement of DA-Raf in TGF-β1-induced EMT in RLE cells, we examined whether DA-Raf participates in the translocation of Smad2 into the nucleus for the transactivation of its target genes leading to EMT. We analyzed the nuclear translocation of mCherry-tagged Smad2 by live cell imaging. Without TGF-β1 treatment, Smad2 was distributed in the cytoplasm but not in the nucleus regardless of whether DA-Raf was knocked down with the *DAraf* siRNA (Fig 6A). Smad2 markedly translocated into the nucleus in the control cells by 60 min after TGF-β1 stimulation, whereas it only marginally translocated into the nucleus in DA-Raf knockdown cells even at 60 min after TGF-β1 stimulation (Fig 6A and 6B; S1 and S2 Movies). Therefore, DA-Raf is indispensable for the nuclear translocation of Smad2, which is required for TGF-β1-induced EMT in RLE cells.

## Discussion

EMT plays crucial roles in a variety of physiological and pathological phenomena, including development, morphogenesis, wound healing, fibrosis, cancer cell invasion, and metastasis [34, 35]. EMT from AEC2s to myofibroblasts is, at least in part, responsible for pulmonary fibrosis [4, 5, 7, 8]. TGF-β, which activates both Smad signaling and non-Smad signaling including the Ras—ERK pathway and PI3K—Akt signaling, critically regulates EMT. Although Smad signaling is indispensable for TGF-β-induced EMT, it remains controversial whether the Ras—ERK pathway is required for or interferes with EMT [22–27]. We have shown here that the Ras—ERK pathway negatively regulates TGF-β-induced EMT in RLE cells. We have also revealed that intrinsic DA-Raf interferes with the TGF-β-induced Ras—ERK pathway and consequently plays an essential role in EMT.

To determine whether the contradictory roles of the Ras—ERK pathway in EMT are due to a difference in the mode and strength of stimulation of the Ras—ERK pathway, we examined the activation pattern of the ERK pathway by TGF-β1 and by TGF-β1 with FGF2 in RLE cells. TGF-β1 stimulation only weakly and transiently activated MEK and ERK immediately after stimulation, whereas stimulation with FGF2 along with TGF-β1 caused strong and sustained activation of MEK and ERK. FGF2 dose-dependently prevented TGF-β1-induced EMT. Therefore, strong and sustained activation of MEK and ERK negatively regulates EMT in RLE cells. This notion is supported by the results that the MEK inhibitor U0126 canceled the inhibitory effect of FGF2 and that the exogenous expression of a constitutively active mutant of H-Ras, B-Raf, or MEK strongly blocked TGF-β1-induced EMT.

Furthermore, these conclusions are in line with the previous findings that the Ras—ERK pathway induced by hepatocyte growth factor (HGF) or FGF1 interferes with TGF-β1-induced EMT in primary cultured AECs and AEC cell lines [26, 27]. In contrast, stimulation with epidermal growth factor (EGF) or transformation with active mutants of H-Ras or C-Raf facilitates TGF-β1-induced EMT in human keratinocytes, canine kidney epithelial cells, and mouse mammary epithelial cells [22–25]. In addition, inhibition of MEK with U0126 prevented EMT in these cells. Therefore, although the Ras—ERK pathway positively regulates TGF-β1-induced EMT in several or many cell types, it negatively regulates in AECs including RLE cells. The molecular mechanisms responsible for this difference remain to be elucidated. However, ERK phosphorylates the linker regions of Smad2/3, which are activated by TGF-β, resulting in suppression of the nuclear translocation of Smad2/3 and of Smad-dependent transcription [36]. On the other hand, the Ras—ERK pathway cooperates with TGF-β to induce Smad-dependent expression of Snail1, which is required for EMT through repressing E-cadherin expression [37]. Thus, these apparently contradictory effects of the Ras—ERK pathway on TGF-β signaling might be differentially exerted between the above different cell types.

Although the Ras—ERK pathway negatively regulates EMT in RLE cells, PI3K—Akt signaling may not affect EMT, because the PI3K inhibitor LY294002 did not reverse the FGF2-induced prevention of EMT. However, the addition of either U0126 or LY294002 together with TGF-β1 severely induced apoptosis. Accordingly, the Ras—ERK pathway and PI3K—Akt signaling activated by TGF-β1, even if they are at low levels of activity, seem to be necessary for preventing apoptosis. Indeed, TGF-β1 has proapoptotic effects, the mechanisms of which differ among different cell types [38]. In contrast, both the Ras—ERK pathway and PI3K—Akt signaling possess antiapoptotic functions [39, 40]. Consequently, one aspect of the physiological significance of the TGF-β1-activated Ras—ERK pathway and PI3K—Akt signaling might be preventing apoptosis that can be induced by TGF-β1.

The knockdown of DA-Raf with the siRNAs facilitated TGF-β1-induced activating phosphorylation of MEK and ERK and interfered with TGF-β1-induced EMT. Thus, intrinsic DA-Raf is essential and sufficient for suppressing the TGF-β1-induced Ras—ERK pathway to cause EMT in RLE cells. However, intrinsic DA-Raf is not sufficient to prevent the high levels of MEK and ERK activities that are induced by FGF2 stimulation or the expression of constitutively active H-Ras, B-Raf, or MEK, to bring about EMT. In other words, the high levels of MEK and ERK activities are beyond the limit that intrinsic DA-Raf can suppress. DA-Raf knockdown impaired the TGF-β1-induced nuclear translocation of Smad2. Thus, DA-Raf plays an essential role in the nuclear translocation of Smad2, which in turn induces transcription of several genes for EMT, by suppressing the Ras—ERK pathway.

There are several proteins that negatively regulate the Ras—ERK pathway. These include Sprouty/Spred proteins [41, 42], RKIP [43, 44], and IMP [45, 46], in addition to DA-Raf. Both Sprouty and RKIP bind to Raf to inhibit the ERK pathway by preventing the binding of MEK. Sprouty also obstructs the growth factor receptor—Ras—ERK pathway by interacting with distinct proteins, depending on the signaling. In contrast, IMP uncouples signal transduction from Raf to MEK by inactivating the scaffold protein KSR. Among them, conditional deletion of Sprouty in lens epithelial cells increases ERK phosphorylation, elevates TGF-β signaling, and promotes TGF-β-induced EMT [47]. Conversely, overexpression of Sprouty in lens epithelial cells suppresses TGF-β-induced signaling and EMT. On the other hands, the effects of RKIP and IMP on TGF-β-induced EMT remain to be elucidated. The effect of Sprouty on TGF-β-induced EMT is inconsistent with that of DA-Raf, which is clarified in this study. It is necessary to examine whether this inconsistency stems from the difference in the regulatory mechanisms between DA-Raf and Sprouty or the difference in cell types used.

**Fig 7:**
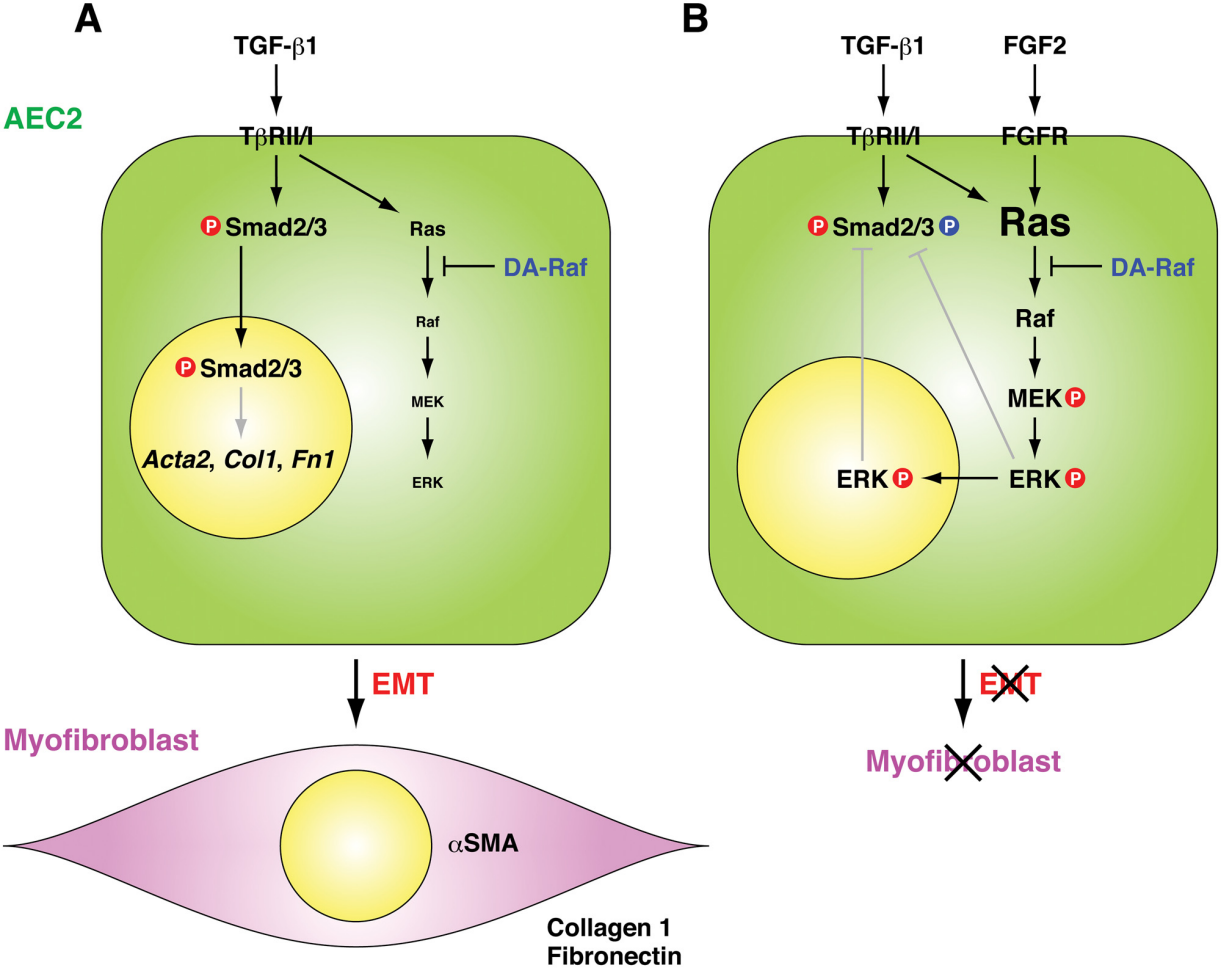
Postulated mechanisms of TGF-β1-induced EMT in RLE cells and the essential function of DA-Raf for EMT. (A) TGF-β1-induced Smad2/3 signaling is essential for EMT from RLE cells to myofibroblasts expressing αSMA, collagen 1, and fibronectin. TGF-β1-induced Ras activity is weak, and intrinsic DA-Raf is sufficient to suppress the ERK pathway by binding to activated Ras. (B) FGF2 stimulation (as well as FGF1 or HGF stimulation) or overexpression of the constitutively active Ras, Raf, or MEK intensifies the activity of the Ras—ERK pathway activated by TGF-β1. Intrinsic DA-Raf is not sufficient to overcome the strong Ras—ERK pathway activity. Activated ERK might interfere with the nuclear translocation of Smad2/3, which is required to induce EMT.

Fig 7 summarizes postulated mechanisms of TGF-β1-induced EMT in RLE cells and the essential role of DA-Raf in EMT. TGF-β1-induced Smad2/3 signaling is required for EMT to convert RLE cells into myofibroblasts expressing αSMA, collagen 1, and fibronectin. TGF-β1-induced Ras activity is weak, and intrinsic DA-Raf is enough to suppress the ERK pathway by binding to activated Ras. In contrast, FGF2 stimulation (as well as FGF1 or HGF stimulation [26, 27]) or overexpression of constitutively active Ras, Raf, or MEK enhances the activity of the TGF-β1-induced Ras—ERK pathway. Intrinsic DA-Raf is not sufficient to overcome the high activity of the Ras—ERK pathway. Consequently, activated ERK interferes with Smad2/3 function that induces EMT. This interference may be achieved directly by phosphorylation of the linker regions of Smad2/3 [27, 36] or indirectly by inducing expression of Smad7, an inhibitor of TβRI—Smad2/3 interaction, and transport of Smad7 and Smurf1, an E3 ubiquitin ligase that causes Smad degradation, from the nucleus to the cytoplasm [26]. Thus, the nuclear translocation of Smad2/3 and subsequent Smad-dependent transcription are prevented. Accordingly, DA-Raf serves as a guardian of TGF-β1-induced EMT by antagonizing the Ras—ERK pathway in at least RLE cells.

TGF-β1-induced EMT in AEC2s is generally considered to be responsible for pulmonary fibrosis [9–13]. Therefore, elucidation of signaling mechanisms that drive TGF-β1-induced EMT in AEC2s may lead to the development of therapeutic strategies to attenuate fibrogenesis. Since DA-Raf does not directly affect Smad2/3 signaling but suppresses the TGF-β1-induced non-Smad Ras—ERK pathway, the information obtained in this study might provide ideas to treatment for pulmonary fibrosis without loss of the other important functions of TGF-β1 in maintaining homeostasis. Furthermore, it is intriguing to examine whether DA-Raf also participates in TGF-β-induced EMT in development or tumor cell invasion. These examinations might develop the notion that DA-Raf is extensively involved in the regulation of EMT.

## Materials and Methods

### Cell culture and EMT induction

Rat AEC2 cell line RLE-6TN (RLE) cells [48] were obtained from American Type Culture Collection (ATCC). RLE cells were cultured in Dulbecco’s-modified Eagle’s medium/Nutrient F-12 Ham (DMEM/F12) (Sigma) containing 10% FBS and subcultured every 2 d. To induce EMT, 1 10^4^ cells/cm^2^ were cultured in DMEM/F12 containing 1% FBS for 24 h and then stimulated with 0.5 ng/ml recombinant human TGF-β1 (R&D Systems) dissolved in 4 mM HCl containing 0.1% bovine serum albumin for 48 h. To assess the effect of FGF2 on EMT, cells were stimulated with 0.5 ng/ml TGF-β1 and 10–100 ng/ml recombinant human FGF2 (Wako) with 100 μg/ml heparin. To analyze signaling pathways, cells were pretreated with 2–10 μM U0126 (Promega) or 10 μM LY294002 (Promega) for 30 min and then stimulated with 0.5 ng/ml TGF-β1 alone or in combination with 50 ng/ml FGF2.

### cDNA cloning, plasmid construction, and transfection

Mouse *Braf*, *Map2k1* (MEK1), and *Smad2* cDNAs containing the entire coding regions were cloned from mouse brain mRNA by reverse transcription (RT)-PCR using Omniscript Reverse Transcriptase (Qiagen) and Phusion High-Fidelity DNA polymerase (Thermo Fisher Scientific). The primer sets used are indicated in S1 Table.

These cDNAs were digested with *Xho*I, *Bam*HI, and *Eco*RI, respectively, and subcloned in pBluescript II vector (Agilent Technologies). Constitutively active B-Raf(V637E) and MEK1(S218D/S222D) cDNAs were constructed by introducing point mutations into wild-type cDNAs in the vector by PCR. The primer sets used are indicated in S2 Table.

These PCR products were self-ligated. *Braf* and *Map2k1* cDNA fragments excised with *Xho*I and *Bam*HI, respectively, were inserted into pEGFP-C1 vector (Clontech) in frame with the EGFP-tag. *Smad2* cDNA fragment excised with *Eco*RI was inserted into pmCherry-C1 vector (Clontech) in frame with the mCherry-tag. pEF-BOS/Myc-H-Ras(G12V) was constructed as described previously [28].

The recombinant plasmids were transfected to RLE cells with Lipofectamine LTX mixed with Plus Reagent (Life Technologies).

### RNAi

Stealth RNAi siRNA duplexes (Life Technologies) were used to knock down rat DA-Raf. The target sequences of the siRNAs were:

- *DAraf* siRNA1: 5´-CCCTGACAGATTATACTTTCGTTTA-3´;
- *DAraf* siRNA2: 5´-GCATGGGACTGTGGGATCATTGGTA-3´;
- control siRNA: 5’-CCCACATAGTATTGACTTTGTGTTA-3’.

The siRNAs were mixed with Lipofectamine RNAiMax Reagent in OPTI-MEM (Life Technologies) and transfected to RLE cells at a concentration of 10 nM.

### Immunoblotting

To detect αSMA, RLE cells were washed with ice-cold PBS and lysed with the SDS sample buffer. For the analysis of signaling proteins, washed cells were lysed with a lysis buffer (1% Nonidet P-40, 50 mM Tris-HCl, pH7.5, 100 mM NaCl, 5% glycerol, 1 mM dithiothreitol, 0.1 mM phenylmethylsulfonyl fluoride, 10 μg/ml leupeptin, 1 μg/ml pepstatin A, 10 mM Na_3_VO_4_, and 10 mM NaF). The lysates were cleared by centrifugation and treated with the SDS sample buffer. The samples were subjected to SDS-PAGE, transferred to PVDF membranes, and analyzed by immunoblotting as described previously [49]. Primary antibodies used were anti-β-tubulin mouse monoclonal antibody (mAb) E7 (Developmental Studies Hybridoma Bank, DSHB), anti-αSMA mouse mAb 1A4 (Sigma), anti-E-cadherin rabbit mAb (Cell Signaling Technology), anti-MEK1/2 rabbit polyclonal antibody (pAb), anti-phospho-MEK1/2 (Ser217/221) rabbit pAb, anti-ERK1/2 rabbit pAb, anti-phospho-ERK1/2 (Thr202/Tyr204) mouse mAb (Cell Signaling Technology), anti-A-Raf rabbit pAb (Santa Cruz Biotechnology), and anti-DA-Raf rabbit pAb [28]. The blotting bands were detected with a ChemiDoc MP system (Bio-Rad). The band intensity was densitometrically analyzed by using ImageJ software (NIH).

### Coimmunoprecipitation assay

The anti-DA-Raf pAb was coupled to Protein A Sepharose 4 Fast Flow (GE Healthcare). RLE cells were lysed with the lysis buffer, and the lysates were incubated with the antibody-coupled Sepharose for 60 min at 4°C [50]. After thorough washing with the lysis buffer, bound proteins were dissociated with the SDS sample buffer and detected by immunoblotting with anti-Ras mouse mAb RAS10 (Millipore).

### Fluorescence microscopy

RLE cells were washed with PBS, fixed with 4% paraformaldehyde in 0.1 M Na-PO_4_ (pH7.4) for 15 min, and permeabilized with 0.1% Triton X-100 in PBS for 5 min. They were incubated with Cy3-conjugated anti-αSMA mouse mAb (Sigma), anti-Myc-tag rabbit pAb (Cell Signaling Technology) for 1 h at room temperature, or anti-GFP rabbit pAb (Medical and Biological Laboratories) overnight at 4°C. The cells were then incubated with Alexa Fluor 488-conjugated anti-rabbit goat IgG (Life Technologies) to detect Myc-tag and GFP. The nuclei were stained with Hoechst 33258 (Life Technologies). The specimens were observed with a Carl Zeiss Axioskop microscope equipped with a CoolSNAP cf CCD camera (Photometrics) operated with Openlab software (PerkinElmer). αSMA-positive and -negative cells were determined by setting a threshold intensity with ImageJ software and counted.

### Real-time PCR

Total RNA was extracted from RLE cells with TRIzol Reagent (Life Technologies) and quantitated with NanoDrop (Thermo Fisher Scientific). Each RNA (2.5 μg) was reverse-transcribed into cDNA with SuperScript VILO cDNA Synthesis Kit (Life Technologies). Real-time PCR was performed with Applied Biosystems 7300 Real-Time PCR System by using SYBR Green I master mix (Life Technologies) as described previously [51, 52]. The primer sets used are indicated in S3 Table.

### Live cell imaging

RLE cells were transfected with the recombinant plasmid pmCherry-C1/Smad2 by electroporation using NEPA21 (Nepagene) and plated on 35-mm glass base dishes. They were transfected with the control or *DAraf* siRNA 12 h after the electroporation. The cells were shifted to DMEM/F12 containing 1% FBS 24 h later and cultured for more 24 h. The dishes were placed on the stage of a confocal laser-scanning microscope FV1200 (Olympus) equipped with a CO_2_ incubator. Live cell images were acquired every 24 s for 60 min after addition of 0.5 ng/ml TGF-β1 to the culture medium. Fluorescence intensity of mCherry—Smad2 in the nucleus and cytoplasm at each time point was analyzed with the region measurement tool of MetaMorph software (Molecular Device).

### Statistical analysis

Statistical analysis was conducted with KaleidaGraph software (Synergy Software). Statistical significance of the data from at least three independent experiments was assessed by Student’s *t*-test.

## Supporting Information

S1 Movie Localization of Smad2 in a control siRNA-expressing RLE cell stimulated with TGF-β1. mCherry—Smad2-expressing RLE cells transfected with the control siRNA were stimulated with 0.5 ng/ml TGF-β1 for 60 min.
(MOV)

S2 Movie Localization of Smad2 in a *DAraf* siRNA-expressing RLE cell stimulated with TGF-β1. mCherry—Smad2-expressing RLE cells transfected with *DAraf* siRNA1 were stimulated with 0.5 ng/ml TGF-β1 for 60 min.
(MOV)

S1 Table Primer sets to clone mouse *Braf*, *Map2k1*, and *Smad2* cDNAs. (PDF)

S2 Table Primer sets to introduce point mutations in *Braf* and *Map2k1* cDNAs. (PDF)

S3 Table Primer sets for real-time PCR. (PDF)
