# Supplementary material for: DA-Raf-Mediated Suppression of the Ras—ERK Pathway Is Essential for TGF-β1-Induced Epithelial—Mesenchymal Transition in Alveolar Epithelial Type 2 Cells
Source: PLoS One. 2015 May 21;10(5):e0127888. doi: 10.1371/journal.pone.0127888 (PMC4440819; doi:10.1371/journal.pone.0127888)
Supplement: S1 Table — (PDF) [file pone.0127888.s003.pdf]

S1 Table. Primer sets to clone mouse *Braf*, *Map2k1*, and *Smad2* cDNAs

***Braf* primer sets with an *Xho*I site**

5´-CGCTCGAGAGATGGCGGCGCTGAGTGGCGG-3´

5´-ATGCTGCCTCGAGGGACTGGCTACTTGAAG-3´

***Map2k1* primer sets with a *Bam*HI site**

5´-CTCTTTCCCGGATCCAAGATGCCCAAGAAG-3´

5´-ATTGCTGGATCCTAAAGGCTCAGATGCTGGC-3´

***Smad2* primer sets with an *Eco*RI site**

5´-TGGAATTCTACCTTTGGTAAGAAAATGTCG-3´

5´-AACAGTCCACGGGATCATACATGAATTCTA-3´
