# Supplementary material for: DA-Raf-Mediated Suppression of the Ras—ERK Pathway Is Essential for TGF-β1-Induced Epithelial—Mesenchymal Transition in Alveolar Epithelial Type 2 Cells
Source: PLoS One. 2015 May 21;10(5):e0127888. doi: 10.1371/journal.pone.0127888 (PMC4440819; doi:10.1371/journal.pone.0127888)
Supplement: S2 Table — (PDF) [file pone.0127888.s004.pdf]

S2 Table. Primer sets to introduce point mutations in *Braf* and *Map2k1* cDNAs

B-Raf(V637E)

5´-TGGAGTGGGTCCCATCAGTTTG-3´

5´-CCGAGATTTCTCTGTGGCTAG-3´

MEK1(S218D/S222D)

5´-AACGACTTCGTGGGCACGAGATCCTAC-3´

5´-GGCCATATCGTCAATTAGCTGCCCGCTG-3´
