# Supplementary material for: DA-Raf-Mediated Suppression of the Ras—ERK Pathway Is Essential for TGF-β1-Induced Epithelial—Mesenchymal Transition in Alveolar Epithelial Type 2 Cells
Source: PLoS One. 2015 May 21;10(5):e0127888. doi: 10.1371/journal.pone.0127888 (PMC4440819; doi:10.1371/journal.pone.0127888)
Supplement: S3 Table — (PDF) [file pone.0127888.s005.pdf]

**S3 Table. Primer sets for real-time PCR**

| | |
|---|---|
| **Rat *Acta2*** | |
| | 5´-AGGATGCAGAAGGAGATCACAG-3´ |
| | 5´-CTGGAAGGTAGATAGAGAAGCC-3´ |
| **Rat *Cdh1*** | |
| | 5´-GGAGAAGAAGACCAGGACTTTG-3´ |
| | 5´-GATGAAGTTCCCGATTTCATCAG-3´ |
| **Rat *Actb1*** | |
| | 5´-TATGCCAACACAGTGCTGTCTG-3´ |
| | 5´-CTCAGGAGGAGCAATGATCTTG-3´ |
